# On-fault earthquake energy density partitioning from shocked garnet in an exhumed seismic midcrustal fault

**DOI:** 10.1126/sciadv.adi8533

**Published:** 2024-03-01

**Authors:** Giovanni Toffol, Giorgio Pennacchioni, Luca Menegon, David Wallis, Manuele Faccenda, Alfredo Camacho, Michel Bestmann

**Affiliations:** ^1^ Department of Geosciences, University of Padova, Padova, Italy.; ^2^ Njord Centre, Department of Geosciences, University of Oslo, Oslo, Norway.; ^3^ Department of Earth Sciences, University of Cambridge, Cambridge, UK.; ^4^ Department of Geological Sciences, University of Manitoba, Winnipeg, Canada.; ^5^ Department of Geology, University of Vienna, Wien, Austria.

## Abstract

The energy released during an earthquake is mostly dissipated in the fault zone and subordinately as radiated seismic waves. The on-fault energy budget is partitioned into frictional heat, generation of new grain surface by microfracturing, and crystal-lattice distortion associated with dislocation defects. The relative contribution of these components is debated and difficult to assess, but this energy partitioning strongly influences earthquake mechanics. We use high-resolution scanning-electron-microscopy techniques, especially to analyze shocked garnet in a fault wall-rock, to provide the first estimate of all three energy components for a seismic fault patch exhumed from midcrustal conditions. Fault single-jerk seismicity is recorded by the presence of pristine quenched frictional melt. The estimated value of energy per unit fault surface is ~13 megajoules per square meter for heat, which is predominant with respect to both surface energy (up to 0.29 megajoules per square meter) and energy associated with crystal lattice distortion (0.02 megajoules per square meter).

## Teaser

The earthquake energy is consumed on-fault by frictional heating, fracturing, and crystal lattice straining.

## INTRODUCTION

**Fig. 1.**
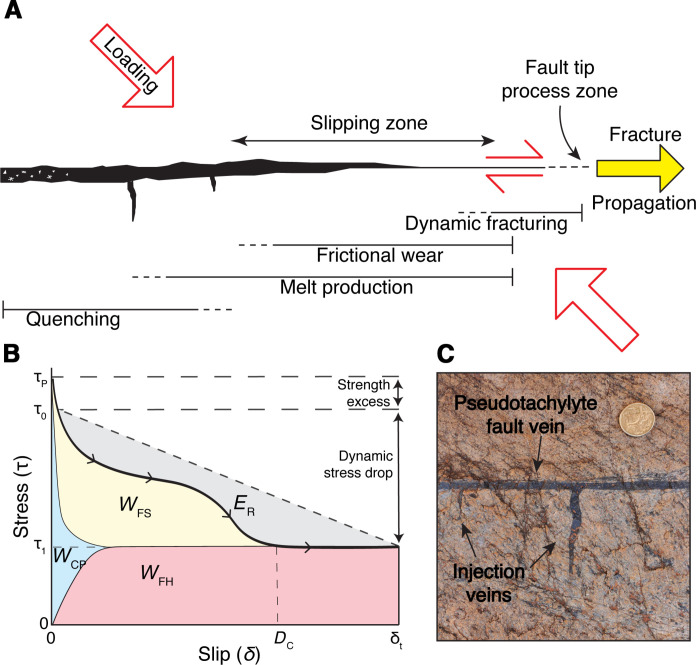
Stages of the earthquake cycle. (**A**) Cartoon illustrating the different processes associated with a fault during the earthquake cycle, including the interseismic loading stage and the coseismic stages of fracture propagation, frictional slip and melting, and the final stage of fault healing by quenching of the pseudotachylyte melt [redrawn after Petley-Ragan *et al.* (*19*); Mancktelow *et al.* (*20*)]. (**B**) Earthquake energy budget per unit fault area for a simplified slip-weakening model represented in the shear stress (τ) versus slip (δ) space. The black curve with arrowheads shows the evolution of stress from the peak strength (τ_p_) to the final value (τ_1_) reached at the critical distance *D*_c_ and maintained until the end of slip (δ_1_). The total released energy is represented by the area below the diagonal dashed line and comprised of the radiated energy (*E*_R_) and the energy dissipated on fault by fracturing, crystal-plastic straining of the minerals and frictional heating (the area below the thick black curve). Energy components are not to scale. Modified after Johnson *et al.* (*16*) to include *W*_CP_. (**C**) Pseudotachylyte fault and injection veins produced by frictional melting during seismic fault slip in a felsic granulite from the hanging wall of the Woodroffe Thrust (25°59′32″S, 132°8′35″E).

Seismogenic environments may account for the buildup of the greatest tectonic differential stresses. High ambient differential stresses are associated with elastic strain accumulated during interseismic periods and partially released during earthquakes. Differential stresses of several hundred megapascals may be achieved by tectonic loading at the brittle-ductile transition in the upper crust (10- to 15-km depth) (*1*). These stresses can reach gigapascal magnitudes in dry rocks of the lower continental crust and in the upper lithospheric mantle (*2*–*6*). Once critically stressed, a fault may yield seismically, partially releasing the accumulated elastic energy in a few seconds. Yielding results in abrupt, large-magnitude, local (near- or on-fault) stress changes during (i) propagation of the earthquake rupture tip at velocities of kilometers per second (*7*) and (ii) frictional fault slip at velocities of meter per second resulting in frictional heating and, eventually, melting of silicate-rich host rocks (*8*–*10*) (Fig. 1A).

These high-stress processes account for dissipation of the largest amount of the released energy of an earthquake as work spent in the fault zone (*E*_FZ_), while a fraction (< 20% of the total energy budget) is radiated as seismic waves (*E*_R_) (*11*). Determining how energy is consumed in the fault zone and what is the contribution of every process is fundamental to properly understand the mechanics of earthquakes (*12*). The energy budget is commonly expressed in terms of work density *W*_FZ_, i.e., the energy spent per area of slipping fault (J/m^2^), and consists of different contributions

$$W_{\text{FZ}}=W_{\text{FH}}+W_{\text{FS}}+W_{\text{CP}}$$ (1)

where *W*_FH_ is the frictional heat, *W*_FS_ is the fracture-surface energy (i.e., the energy consumed to fracture the rock and propagate the rupture), and *W*_CP_ is the energy consumed in crystal-plastic and elastic straining of mineral lattices (Fig. 1B). These quantities, unlike the energy radiated as seismic waves, can only be estimated indirectly during an earthquake. Most of the available estimates of the fault-zone work come from laboratory experiments and interpretations of seismological observations [see review by Cocco *et al.* (*12*)]. Only a few studies have inferred these quantities from local observations along natural seismogenic fault zones (*13*–*16*). Direct estimates of the on-fault energy budget from exhumed seismogenic faults may help to clarify the dynamics of coseismic processes in different structural settings and to bridge field observations and experimental results (*12*).

The geological record of an earthquake can be exceptionally well preserved in exhumed faults that contain pseudotachylytes, which are quenched frictional melts recognized as the product of seismic slip (*9*, *17*–*20*) (Fig. 1C). The microstructures of pseudotachylytes and surrounding fault rocks can capture snapshots of the loading history (*19*, *21*, *22*), as well as the seismic rupture propagation and slip [e.g., fracturing and pulverization (*18*–*20*, *23*, *24*)]. Features like the presence of pulverized minerals, the fault roughness, and the thickness of pseudotachylyte fault veins have been used to investigate earthquake mechanics and obtain mechanical parameters, such as the dynamic friction coefficient, the frictional heat, and the fracture energy. In particular, the study of a single-jerk pseudotachylyte fault vein, well characterized from a (micro-)structural point of view, is useful to associate the measured energy densities to a single event (*15*, *25*). With this approach, uncertainties on the number of earthquakes responsible for the fault structure (*13*, *16*) are avoided, although the information is necessarily local and limited to a small fault patch.

Although evidence of crystal-plastic and elastic effects in fault rocks surrounding pseudotachylytes have been described [e.g., Bestmann *et al.* (*9*)], the contribution of *W*_CP_ in the energy budget has never been properly quantified, as this requires precise measurements of elastic strains and the density of dislocations. High-angular resolution electron backscattered diffraction (HR-EBSD)—a material science technique only recently applied in geosciences—provides a way to measure, with high precision and spatial resolution, the residual strains and dislocations stored in the lattices of minerals (*26*, *27*). Here, we present the first application of HR-EBSD to garnets that were seismically shocked at midcrustal levels. The sluggish dislocation mobility in garnet at the ambient conditions of faulting, the absence of twinning and cleavage, and the high fracture toughness make garnet a suitable candidate to preserve coseismic residual strains and high densities of dislocations. Combined with estimates of *W*_FH_ and *W*_FS_ obtained from the study of the pseudotachylyte and pulverized portions of the host rock, we provide a complete energy budget of the fault-zone work during a single earthquake revealing a precise hierarchy in the contributions of the different work-density components of an earthquake.

## RESULTS

### Sample description

The studied sample (25°59′48″S, 131°39′48″E) is from the hanging wall of the Woodroffe Thrust (Musgrave Ranges, central Australia), a major east-west-striking, gently dipping, crustal-scale mylonitic zone developed at intermediate crustal levels during the Petermann Orogeny (630 to 520 Ma) (*28*, *29*). The thrust juxtaposes upper-amphibolite/granulite-facies rocks of the Fregon Subdomain in the hanging wall with amphibolite-facies granitoids and gneisses of the Mulga Park Subdomain in the footwall (*30*, *31*). Toward the thrust, the Fregon Subdomain contains the largest volumes, worldwide, of tectonic pseudotachylytes (*32*).

**Fig. 2.**
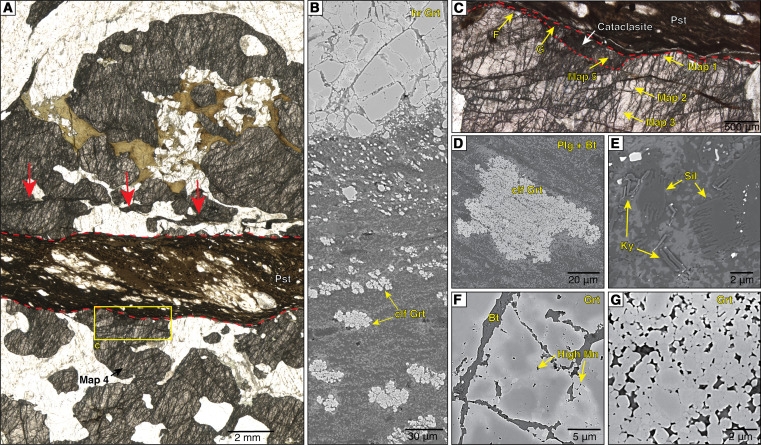
Microstructures. (**A**) Overview of the pseudotachylyte (Pst) vein crosscutting the host rock fractured garnets with quartz (light colored) and biotite (brown-green) inclusions. Red arrows indicate auxiliary sharp zones of cataclasis. The location of the map 4 and the yellow rectangle [enlarged in (C) and including the locations of maps 1 to 3 and 5] are shown. (**B**) Fractured garnet in the pseudotachylyte host rock and cauliflower-shaped garnets (clf Grt) inside the pseudotachylyte with increasing sizes toward the vein core. (**C**) Detail of the host-rock garnet showing locations of maps 1, 2, 3, and 5. (**D**) Cauliflower garnet in the layered plagioclase-biotite matrix of the pseudotachylyte. The matrix has an equant polygonal microstructure, locally with shape preferred orientation of biotite parallel to the flow foliation. (**E**) Sillimanite (Sil) microlites and acicular kyanite (Ky) within a pseudotachylyte crosscutting a peraluminous host-rock layer. Sil microlites overgrow Sil clasts from the host rock. (**F** and **G**) Back-scattered electron images showing details of the fractured and cataclastic garnet close to the pseudotachylyte. Light-gray portions are enriched in Mn. (A) and (C) are visible-light microscope images in plane polarized transmitted light). (B) and (D) to (G) are back-scattered electron images.

The sample, hosting a 3-mm-thick pseudotachylyte vein, is a felsic granulite composed of quartz, plagioclase, K-feldspar, centimeter-sized garnet, and minor biotite and sillimanite. The pseudotachylyte is pristine, without any overprint by solid-state ductile deformation and alteration. The average composition of the pseudotachylyte is, by area, 61% plagioclase (andesine), 27% biotite, and 12% garnet, plus rare K-feldspar and quartz. Clasts, mainly quartz and feldspars, account for 19.4% of the total pseudotachylyte area (fig. S1). The syn-melt flow foliation of the pseudotachylyte, oblique to the boundary (Fig. 2A), is marked by (i) elongated cauliflower-shaped garnet microlites (locally overgrowing clasts of host-rock garnet) of increasing grain size toward the vein center (Fig. 2B) and (ii) a local compositional banding of the fine-grained (<1 μm grain size) biotite-plagioclase (±quartz, ±K-feldspar) matrix (Fig. 2D). Pseudotachylytes from the same set of veins of the studied sample, cutting across sillimanite-rich peraluminous gneisses in the same outcrop, contain abundant microlites of sillimanite and acicular kyanite (Fig. 2E) together with minor andalusite and garnet. This assemblage is observed in other localities along the Woodroffe Thrust (*33*) and constrains the ambient temperature and pressure of seismic faulting to have been around 500°C and 0.5 GPa, respectively (*33*, *34*).

The granulitic almandine-rich (Almandine_0.66_ Pyrope_0.21_ Spessartine_0.08_ Grossular_0.05_) host-rock garnet shows the same intense fracturing on both sides of the pseudotachylyte (Fig. 2 A to C). The microfractures are arranged in two main sets with slightly different orientations in different garnet grains. Most fractures are filled with biotite and, mostly, do not offset the garnet boundary. The biotite-filled fractures crosscut fractures overgrown, and partially healed, by garnet that is enriched in Mn (up to 8 wt % from the 2 to 3 wt % in the host) (Fig. 2F). These healed fractures are especially developed nearby the pseudotachylyte. Here, garnet is ultracataclastic (grain size as small as 0.02 μm in contact with the pseudotachylyte) (Fig. 2G) and the clasts are typically rimmed by new Mn-enriched garnet, resulting locally in a polygonal aggregate with tight, triple grain junctions (Fig. 2F). Similar polygonal aggregates occur along healed microfractures (Fig. 2F) and along sharp auxiliary cataclastic bands, subparallel to the pseudotachylyte vein, across the host granulitic garnet.

### HR-EBSD maps

Five areas (approximately 1900 to 5200 μm^2^) were selected for HR-EBSD mapping. Maps 1 to 4 are in the host-rock garnet at increasing distances (approximately 0, 0.3, 0.6, and 2 mm, respectively) from the pseudotachylyte boundary. Map 5 is within the cataclastic garnet at the contact with the pseudotachylyte (see Fig. 2 and fig. S2 for locations). The five areas were selected after inspection of standard EBSD maps (fig. S2).

**Fig. 3.**
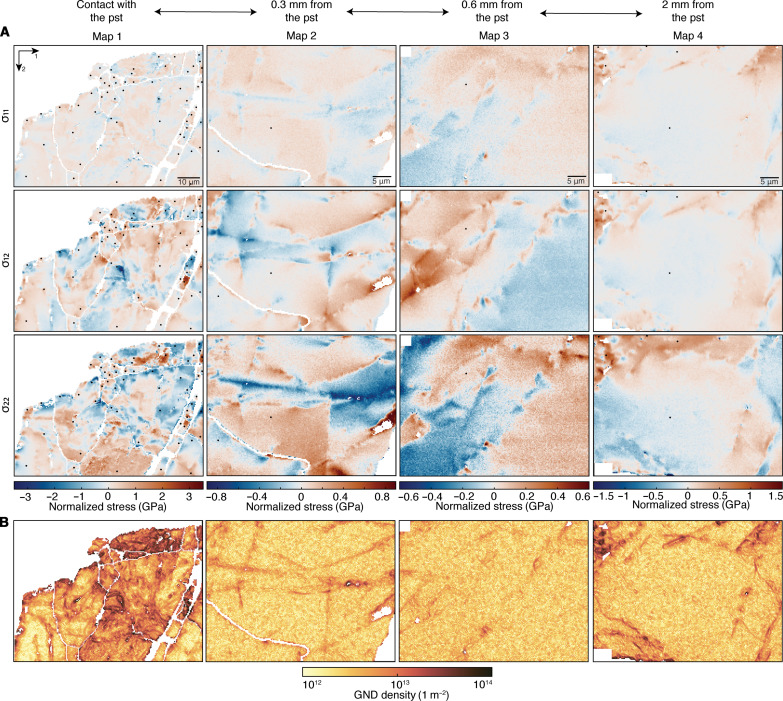
Residual stress heterogeneities and GND densities at increasing distance from the pseudotachylyte fault vein. (**A**) In-plane components of the residual stress. Stress is normalized to the mean stress for each grain (see Methods). White areas are not-indexed pixels or pixels that do not meet the quality criteria (see Methods). Black dots mark the selected reference points. (**B**) GND densities obtained from the measured lattice rotations.

**Fig. 4.**
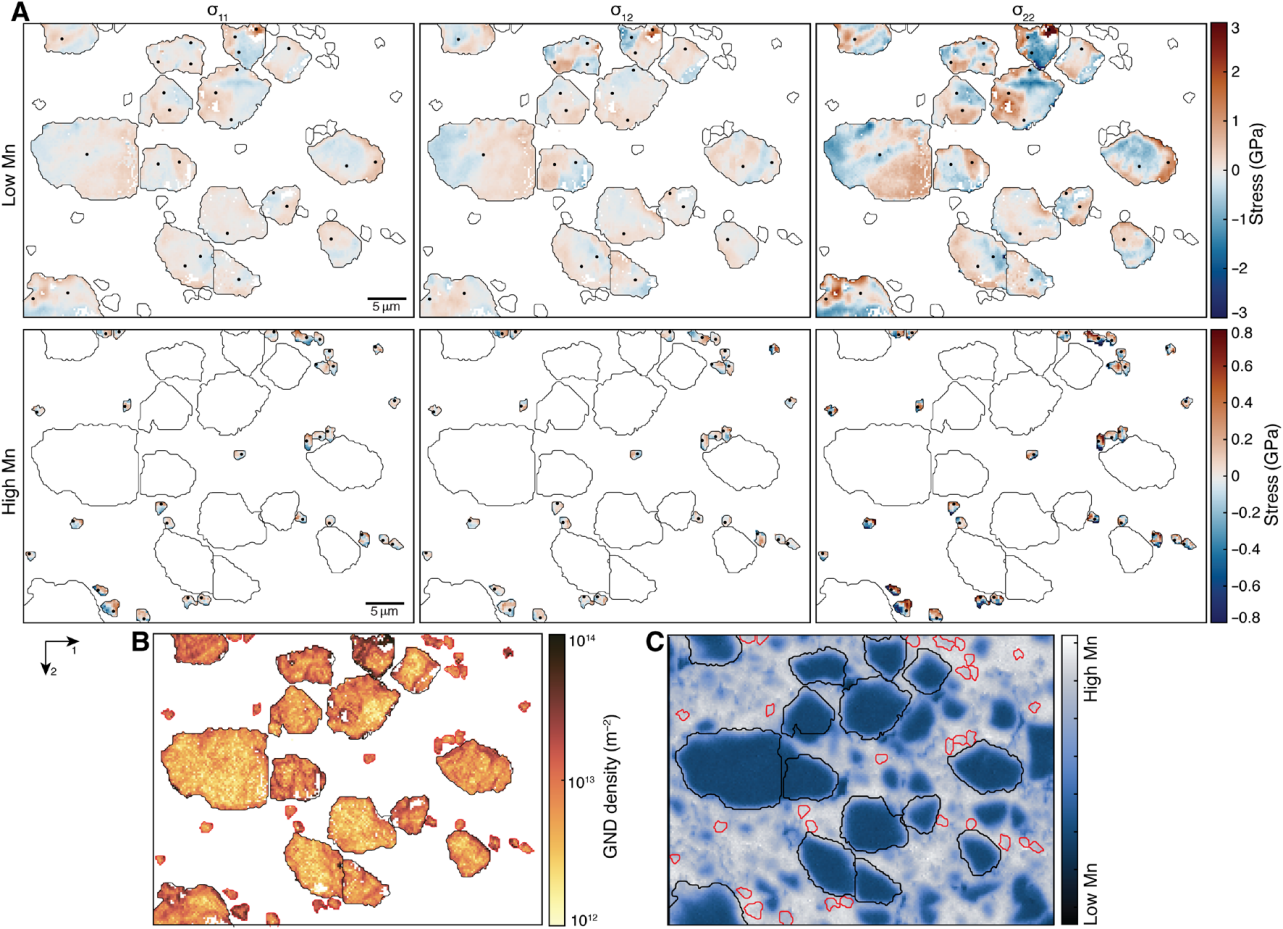
Residual stress heterogeneities and GND densities in the cataclastic domain. (**A**) In-plane components of the residual stress. Stress is normalized to the mean stress for each grain (see Methods). White areas are not-indexed pixels or pixels that do not meet the quality criteria (see Methods). Black dots mark the selected reference points. Results are divided between low-manganese and high-manganese garnets [see (C)]. Only selected grains were analyzed because of the heterogeneous quality of the electron backscattered diffraction (EBSD) patterns. (**B**) GND densities obtained from the measured lattice rotations. (**C**) Chemical map of manganese. Black and red lines mark the boundaries of the low-manganese and high-manganese garnets, respectively.

The estimated in-plane components (i.e., acting in the thin-section plane) of the residual stress tensor and the geometrically necessary dislocation (GND) densities are shown in Figs. 3 and 4. The stress values are relative to the unknown stress state at the selected reference points and therefore provide a measure of the spatial heterogeneity in stress rather than absolute values. To provide an intuitive measure, we normalize the stresses by subtracting the average of each component within each grain/fragment to present stress heterogeneity relative to the unknown average stress state within each area (see Methods).

In maps 1 to 3, the heterogeneity in intragranular in-plane residual stress increases from a few hundreds of megapascals (map 3) to more than 5 to 6 GPa (map 1, σ_12_ and σ_22_) approaching the pseudotachylyte. Maps 2 and 3 show abrupt variations and change in sign of residual stress across straight bands. In map 1, stress heterogeneity occurs over smaller length scales and is more irregularly distributed in space. Map 4, located farther away (approximately 2 mm) from the pseudotachylyte than map 3, exhibits greater stress values than in both maps 2 and 3, and smoother stress changes without sharp straight boundaries of abrupt stress variation. Therefore, map 4 does not fit with the gradient of progressive decrease of residual stress observed from map 1 to map 3. The same spatial gradient observed for the residual stresses is reflected in the GND densities, with the highest values measured close to the pseudotachylyte (map 1), locally up to 3 × 10^14^ m^−2^. With increasing distance, the GND densities decrease to <10^13^ m^−2^ with only local higher values. High GND densities are typically located along the straight boundaries at which the sign of stress switches and, in general, correspond to the areas in the maps where the greatest residual stresses are recorded.

Map 5 (Fig. 4) images a band of cataclastic garnet directly in contact with the pseudotachylyte. The clasts are overgrown by garnet enriched in Mn, with euhedral shapes in the finest micrometric clasts. The larger clasts (5 to 15 μm in diameter) record stress heterogeneity in the range 2 to 4 GPa (σ_12_ and σ_22_), comparable to values measured in map 1. In contrast, the smaller, Mn-enriched garnet clasts are less distorted and exhibit stress heterogeneities of up to 1 to 1.5 GPa. However, measurements in small grains must be considered with caution as measurement points could be influenced by topographic effects near grain boundaries due to sample preparation. The GND densities, similar to the residual stress heterogeneities, are comparable to those measured in map 1.

**Fig. 5.**
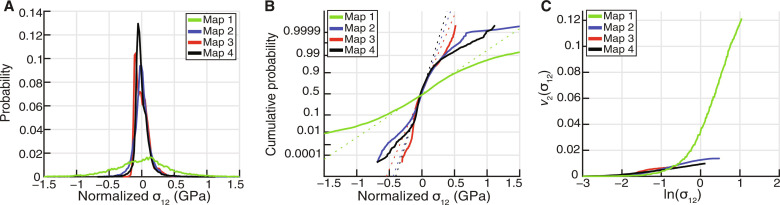
Probability distribution and distribution form analysis of the stress heterogeneities in maps 1 to 4. (**A**) Probability distribution of normalized σ_12_ for the four maps, calculated with a bin width of 20 MPa. (**B**), Normal probability plot of normalized σ_12_ for the four maps. Normal distributions correspond to straight lines (represented as dotted lines). (**C**) Restricted second moments of the normalized σ_12_ for the four maps. Straight lines represent probability following *P*(σ) ∝ |σ^−3^|.

Figure 5 presents probability distributions of the normalized σ_12_ in-plane stress component and characterizes the shape of the high-stress tails of the distributions (also fig. S3). The stress distributions are broadest in map 1, where the greatest residual stress heterogeneity is measured. Normal probability plots, in which the cumulative-probability axis is scaled such that a normal distribution is represented by a straight line, are useful to visualize the shape of the high-stress tails of the distributions (Fig. 5B). The central, low-stress portion of each distribution follows a straight line whereas the high-stress tails deviates from the linear trend indicating that they do not follow the same normal distribution. The deviation is most accentuated in map 1. The shapes of the tails are characterized by plots of the restricted second moments (υ_2_) of the stress distributions versus ln(σ) (Fig. 5C and fig. S3; see Methods for details). At high stresses the distributions follow straight lines, meaning that *P*(σ_12_) ∝ |σ_12_^−3^|, with map 1 having a steeper slope extending to higher stresses. The inverse cubic form is expected if the stress field is determined by the presence of dislocations, and the gradient of the curve is proportional to the total dislocation density (*35*). We also subset each map based on the corresponding GND densities at each pixel and computed the restricted second moments of the stress distributions for each subset (fig. S4). Subsets with greater GND densities exhibit steeper slopes of the straight portions of the curves and in general extend to higher stresses. Only map 3 shows similar curves for all the subsets.

### Clast size distribution

**Fig. 6.**
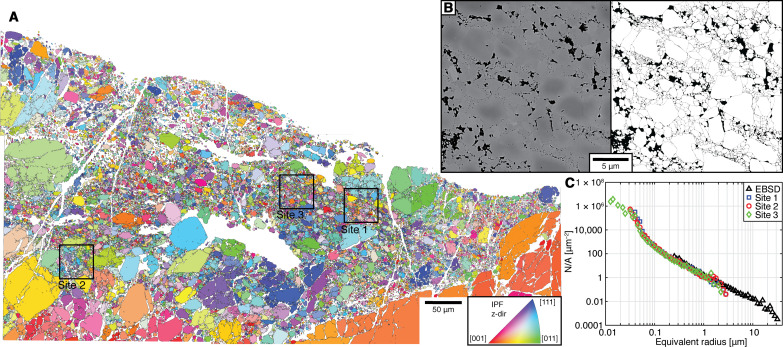
Grain-size analysis for the cataclastic domain. (**A**) Mosaic of four electron backscattered diffraction (EBSD) maps color-coded by the mean orientation of every grain according to the inset inverse pole figure. (**B**) High-resolution backscattered electrons (BSE) image of a fine-grained portion (site 3 marked in the EBSD map) and segmented garnet grains (white). (**C**) Area-weighted (cumulative number of grains divided by the total area of the fragments, N/A) clast-size distributions determined from EBSD data and the three high-resolution BSE images plotted in a log-log space.

The clast-size distribution was measured in the cataclastic domain where map 5 was acquired. Large EBSD maps (Fig. 6A), covering most of the domain, allow clasts down to a size of 0.22 μm to be well indexed and measured. The remaining nonindexed portions in the EBSD maps (~30%) correspond to phyllosilicates, holes, and the finest-grained garnet clasts. High-resolution backscattered-electron (BSE) images were used to measure the clast-size distribution of these finer-grained (down to 20 nm in equivalent radius) portions (Fig. 6B and fig. S5). The clast-size distributions are plotted in log-log diagrams and fitted with polylines with decreasing slopes (−*D*) at decreasing clast size (fig. S6). The clast-size distribution estimated by EBSD analysis shows *D* > 2 for clast sizes >0.7 μm and *D* = 1.15 for the smaller clasts (0.7 to 0.22 μm). The three clast-size distributions estimated from BSE images are consistent among each other and show the following: (i) *D* = 2 to 2.4 for clast size > ~0.3 μm; (ii) *D* = 1 to 1.24 for clast size 0.3 to 0.08 μm; and (iii) an almost flat distribution for the smallest clast sizes. The clast-size distributions normalized by the total area covered by each clast class (Fig. 6C) show a good match over the range of overlapping equivalent radii.

## DISCUSSION

### Lattice damage by dynamic rupture propagation

The HR-EBSD maps reveal high GND densities and high magnitudes of stress heterogeneity stored in the lattice of garnet crystals damaged during an earthquake. The zone of high stress heterogeneity and high GND density extends only a few millimeters in thickness from the pseudotachylyte boundary. Heterogeneity in residual stresses of as much as 5 to 6 GPa at the contact with pseudotachylyte decrease to a few hundred megapascals over a distance of less than 1 mm. Such an extreme and localized gradient of GND density and residual stress heterogeneity well match the gradient of the dynamic stress field surrounding the tip of an earthquake rupture propagating at a velocity close to the Rayleigh velocity (*7*).

Elastic straining of the garnet lattice took place associated with pulverization. Extreme comminution occurred mainly at the contact with the pseudotachylyte, where the greatest residual stresses are stored, but also along auxiliary planes. Pulverization is common in the damage zone of upper-crustal faults (*36*, *37*) but has been also described from high-pressure natural and experimental pseudotachylytes. Austrheim *et al.* (*38*) interpreted pulverized garnets within a few millimeters distance from a pseudotachylyte to be the result of fault slip due to changes in stress between compression and extension. Coseismic pulverization in the process zone was described in garnets and pyroxenes from lower-crustal rocks (*19*, *20*), and in olivine, clinopyroxene and plagioclase in ophiolites exhumed from an intermediate-depth subduction environment (*39*). Pulverization was also reproduced experimentally at high-pressure conditions (*40*) and interpreted as the result of the high tensile stresses at the tip of a propagating fracture. Therefore, we infer that garnet comminution and fracturing and the associated high GND densities and residual stresses were produced during the propagation of the earthquake rupture tip. This interpretation is reinforced by the fact that fracturing predates melting along the fault plane as pulverized material is crosscut by pseudotachylyte injection veins and included as clasts in the pseudotachylyte [as also interpreted by Mancktelow *et al.* (*20*)]. The abrupt variations in magnitude and sign of stress across sharp bands without offset are also features consistent with pulverization, because the sharp bands are likely healed or incipient cracks. This interpretation is also consistent with features in map 5, where the later high-Mn garnets record lower GND densities than the original low-Mn garnet clasts in the same structural position.

The residual stresses and GND distributions in the garnet likely resulted from the dynamic stress field around the tip of a mode II fracture propagating close to the Rayleigh-wave velocity. Following Freund (*41*), the stress field can be calculated as

$$\sigma_{\mathrm{ij}}=\left(\frac{K_{\text{II}}}{\sqrt{2\pi r}}\right)*\sum_{\mathrm{ij}}^{\text{II}}\left(\theta ,v\right)$$ (2)

and its magnitude is controlled by the stress intensity factor *K*_II_, the fracture-propagation velocity *v*, the distance from the tip, *r*, and the angle θ (see Methods). Assuming typical material properties for middle-crustal rocks and a fracture energy of 10^6^ J/m^2^, in the range of values proposed for earthquake ruptures (*12*), calculations show that the short-lived (a few microseconds), high-stress pulse can exceed 10 GPa and stresses substantially drop over a few millimeters distance from the slipping plane (fig. S7). The distance of host-rock domains from the original earthquake rupture plane cannot be precisely established, because the process zone associated with the earthquake rupture propagation was reworked by frictional wearing and melting during seismic slip. However, the measured domains of maps 1 to 3 within the host-rock garnet still preserve a GND-density gradient compatible in shape with the stress field predicted nearby the rupture plane and with the sharp spatial gradient normal to the fracture. Map 4 does not strictly follow the gradient recorded by maps 1 to 3. This deviation may reflect local variations of the propagating rupture stress field, effects of secondary fractures, or interactions among different grains.

### Preservation of GNDs and residual stress

The restricted second moments of the probability distributions of stress shows that at higher stresses the distributions have the form *P*(σ_ij_) ∝ |σ_ij_^−3^|. This distribution indicates that the residual stress heterogeneity with the greatest magnitude results from the stress fields around dislocations (*35*, *42*, *43*). High dislocation densities were described from olivine and quartz that have been experimentally deformed at coseismic conditions and not subsequently annealed (*44*, *45*). High dislocation densities were also observed in quartz in host rock flanking upper-crustal pseudotachylytes and were interpreted to result from high coseismic stress (*9*, *46*). Within quartz, dislocations are recovered during the high thermal pulse in contact with the pseudotachylyte (*9*). In contrast, the increase in GND density and residual stress toward (and up to the contact with) the pseudotachylyte is preserved within the garnet analyzed here, which we interpret to be due to the more sluggish mobility of dislocations in garnet than in quartz at the ambient deformation conditions (*47*). The seismically induced dislocation arrays in shattered quartz described by Bestmann *et al.* (*9*) were completely erased by annealing during the thermal transient associated with frictional heating and melting (see their figure 6). However, it is of note that these dislocation arrays are preserved about a hundred micrometers away from the pseudotachylyte despite seismic faulting occurring at ambient conditions close to the low-temperature limit of crystal plasticity in quartz. These observations suggest that earthquake-induced dislocations can be preserved after formation through the exhumation path to Earth’s surface even at conditions close to the onset of crystal plasticity. This point is even more valid for garnet that typically deforms by crystal plasticity at higher temperature than quartz and well above the ambient condition of the pseudotachylyte formation studied here (*47*, *48*).

The lack of recovery of GNDs in the studied sample is responsible for the measured residual stresses. The absence of any other deformation, metamorphism and interaction with fluids after the earthquake allowed preservation of the crystal-lattice distortions produced during the earthquake-rupture propagation.

### Energy budget of an earthquake

**Fig. 7.**
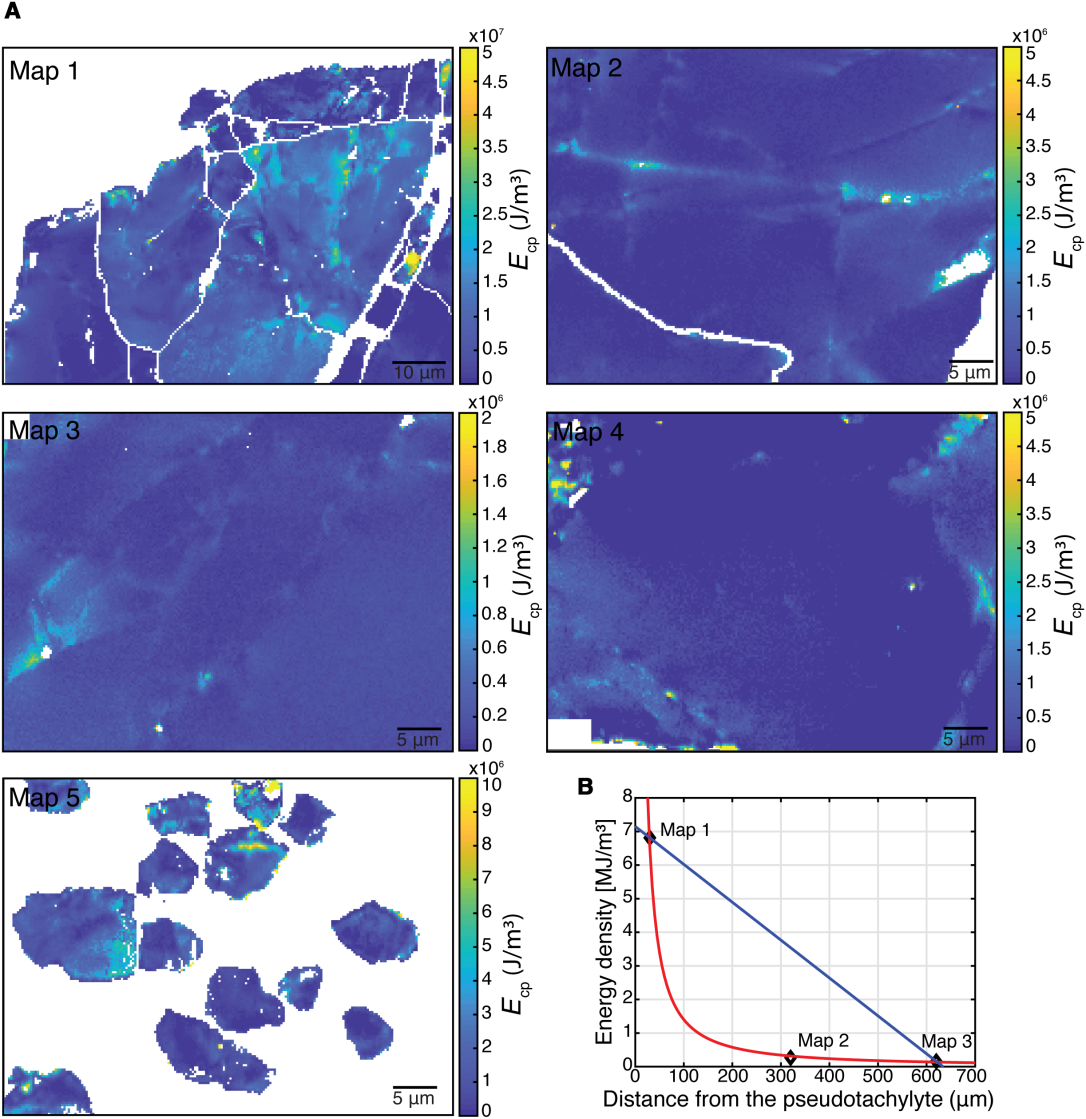
Energy densities stored as dislocations and their strain fields. (**A**) Maps of energy density stored in the lattice of the garnet, calculated from HR-EBSD data shown in Figs. 3 and 4. (**B**) Mean energy density as a function of distance from the pseudotachylyte margin for maps 1 to 3. Red and blue curves show the power-law and linear interpolation curves.

The energy stored in the lattice of the host-rock garnets, in terms of GND densities and associated residual elastic strains, corresponds to the energy necessary for dislocation generation (*49*). Thus, the energy density can be used to quantify the damage imparted to the rock during the propagation of the earthquake rupture at the studied patch of seismic fault. The total energy density stored in the lattice (*E*_cp_) can be calculated as the sum of the energy density stored as elastic strain (*E*_st_) and the energy density stored as dislocation cores (*E*_disl_), which are computed as

$$E_{\text{st}}=1/2\left(\sigma_{11}\varepsilon_{11}+\sigma_{22}\varepsilon_{22}+2\sigma_{12}\varepsilon_{12}\right)$$ (3)

and

$$E_{\text{disl}}=\rho_{\text{GND}}\left(Gb^{2}\right)/\left(2\pi \right)$$ (4)

respectively, with *G* the isotropic shear modulus (100 GPa), *b* the length of the Burgers vector (1 nm) (*50*), and ρ_GND_ the density of GNDs measured with HR-EBSD at every pixel of the map. The energy estimates are shown in Fig. 7. We considered average values of each map as the mean energy densities at the specific distance from the pseudotachylyte (6.8 × 10^6^, 2.6 × 10^5^, 1.6 × 10^5^, and 9.2 × 10^4^ J/m^3^ at 0, 0.3, 0.6, and 2 mm of distance from the pseudotachylyte respectively; 1.5 × 10^7^ J/m^3^ in the large clasts of the cataclastic portion).

To convert *E*_cp_ (J/m^3^) into *W*_CP_ (J/m^2^), we computed the interpolating function of the energy densities collected in the same garnet for the maps 1 to 3 and then integrated it across the investigated garnet over a distance of approximately 1.2 mm. *W*_CP_ estimated from the host-rock garnet varies between 1.5 × 10^3^ and 2.4 × 10^3^ J/m^2^ depending on whether a linear or power-law interpolation is considered, respectively. However, consistent with the observations of Pittarello *et al.* (*15*), the breakdown work recorded in the host rock adjacent to the pseudotachylyte is likely a minor component of the real work density, as most of the highly strained and comminuted material was extensively consumed during the frictional melting. We therefore calculate the bulk energy by extrapolating the maximum estimated value of *E*_cp_ (in map 1) over the total thickness of the pseudotachylyte measured on the sample with the awareness that this estimate still provides an underestimate of the actual energy. Under this assumption, and also assuming that the pseudotachylyte thickness is representative for the studied seismic fault, we estimate 2 × 10^4^ J/m^2^ as bulk *W*_CP_ value. A further complication is related to the intrinsic uncertainties of the HR-EBSD measurements (see Methods).

*W*_FS_ is calculated from the clast-size distributions measured in the cataclastic domain. As for the calculation of *W*_CP_, the clast-size distribution measured in contact with the pseudotachylyte has been considered representative of the material consumed by frictional melting within the pseudotachylyte and therefore extrapolated over the whole pseudotachylyte thickness. Although the clast-size distributions cover only three orders of magnitude and show at least one clear variation in slope, we consider the real clast-size distribution as fractal, with a value of *D* = 2.1 that best approximates the slope of the clast-size distribution for clast sizes >0.1 μm and extrapolated over the entire dimensional range (0.02- to 20-μm clast equivalent radii). The estimated values of *W*_FS_ for this fractal number and grain-size range vary between 1.3 × 10^4^ and 2.9 × 10^5^ J/m^2^ (Methods). These values fall on the low side of other measurements reported for natural faults in the literature (1 × 10^4^ to 1 × 10^7^ J/m^2^) (*13*–*16*). Our upper value of *W*_FS_ is similar to the estimate of Pittarello *et al.* (*15*), who also considered a single-jerk, pseudotachylyte-bearing, upper-crustal fault within tonalite in their study and used the same approach of extrapolating the locally measured clast-size distribution of host-rock plagioclase over the whole pseudotachylyte thickness.

Frictional heat was calculated considering the energy consumed in rock heating, melting and further heating of the melt (*25*) (Methods). We assumed a maximum temperature of the melt of 1450°C, based on the observation that plagioclase preferentially melted whereas quartz persisted as clasts of disaggregated fragments. For a 3-mm-thick pseudotachylyte fault vein made of plagioclase, biotite and garnet and with ~20% of clasts, the energy dissipated by frictional heating *W*_FH_ is approximately 13 × 10^6^ J/m^2^, on the same order of magnitude as that estimated by Pittarello *et al.* (*15*) for their pseudotachylyte-bearing fault. In summary, *W*_FH_ is two orders of magnitude greater than *W*_FS_ and three orders of magnitude greater than *W*_CP_.

The high dynamic stresses at the tip of a propagating earthquake rupture induce, together with host-rock fracturing and cataclasis, intense elastic deformation and GNDs in the lattice of host-rock minerals. These latter microstructures can escape annealing along the exhumation path to Earth’s surface. We have quantified the energy component associated with the coseismic crystal-plastic damage produced by the earthquake rupture and have estimated the other components of breakdown work spent on the fault during an earthquake from an exhumed pseudotachylyte-bearing midcrustal fault providing a complete on-fault energy budget. The estimates establish a clear hierarchy in the energy-density partitioning of breakdown work. Heat accounts for the greatest part of the energy consumed on fault, as it is two and three orders of magnitude greater than the fracture energy and the crystal-plastic energy, respectively. The local accumulation of crystal-plastic energy densities in the lattice of faulted minerals, here exceptionally preserved in the wall-rock garnet, may eventually play a role in postseismic physical-chemical processes on the fault plane, e.g., by hampering postseismic creep due to local strain hardening or by promoting metamorphic re-equilibration providing energy expendable to overstep reactions.

## MATERIALS AND METHODS

### Sample preparation and data acquisition

The thin section was mechanically polished and finished with a colloidal-silica suspension to ensure a damage-free surface required for HR-EBSD.

BSE imaging was performed with a TESCAN Solaris field emission gun scanning electron microscope (FEG-SEM) at the Department of Geosciences, University of Padova, equipped with a midangle BSE detector. Working conditions were 5-kV acceleration voltage, 300-pA beam current, and 4-mm working distance. Electron backscattered diffraction (EBSD) and energy-dispersive x-ray spectroscopy (EDS) data were acquired with a Hitachi SU500 FEG-SEM equipped with Dual Bruker Quantax XFlash 30 EDS system and a Bruker e-Flash HR EBSD system at the Goldschmidt Laboratory, Department of Geosciences, University of Oslo. Preliminary EBSD maps and maps for HR-EBSD were acquired and indexed with Esprit software and processed with the MTEX toolbox (*51*) version 5.6.1 in MATLAB.

EBSD maps were acquired with an acceleration voltage of 15 kV, working distance of 18 mm, sample tilt of 70° and detector tilt of 1.9°. Preliminary EBSD maps have step sizes in the range 0.8 to 0.3 μm. EBSD maps for HR-EBSD analysis have step sizes of 0.25 and 0.41 μm. For HR-EBSD, maps were acquired saving the patterns at a resolution of 1200 × 1200 pixels and subsequently processed with the commercial software CrossCourt Rapide 4.5.

EBSD maps for measurements of clast size were acquired with a TESCAN Solaris equipped with an Oxford Instruments Symmetry EBSD detector and indexed with Oxford Instruments AZtec software. Acquisition conditions were 20 kV of acceleration voltage, 10 nA of current, 15 mm of working distance, and a step size of 0.15 μm.

Chemical data were acquired with a Cameca SX100 electron probe microanalyzer equipped with five WDS spectrometers at the Department of Geosciences, University of Oslo. Conditions of acquisition were 15-kV acceleration voltage and 15-nA beam current.

### HR-EBSD analysis

HR-EBSD is based on cross-correlation between each EBSD pattern within grain and a pattern from the same grain chosen to be a reference. From the measurement of shifts in small regions of interest within the patterns, the rotation and strains of the crystal lattice can be measured with precision on the order of 10^−4^ (*26*). A comprehensive review of HR-EBSD applied to geological materials is provided by Wallis *et al.* (*27*).

EBSD maps were filtered (indexed bands >6) before the cross-correlation, to exclude bad-quality patterns from the analysis. Patterns acquired along fractures or close to surface imperfections (as identifiable from band-contrast maps) were manually checked and deleted if blurred or showing superposition of two different patterns. A subsequent cleaning stage was performed after the first cross-correlation step to exclude other bad patterns previously neglected and subsequently evidenced by high values of mean angular error and high-resolution kernel average misorientation (HR-KAM). A reference point for every grain in the map was automatically selected by the software, initially on the basis of the KAM values from the initial EBSD and, for subsequent cross-correlation stages, on the HR-KAM obtained after the first pass of cross-correlation. Where needed, other reference points were added manually to improve the quality of the analysis. If more than one reference point was used, the results were automatically referred to the first reference point considered for each grain in the map by adjusting the others on the basis of the differences in the measurements obtained in the overlapping regions for the respective reference points. Cross-correlation of each pattern with the reference pattern was performed considering 40 to 100 regions of interest (256 × 256 pixels in size) per pattern and applying the robust fitting procedure (*52*). Cross-correlation is performed in Fourier space, and low- and high-pass filters were applied to the patterns to reduce the noise level of the measurements. An effective pixel size of 18.23 μm (calibrated on an unstrained Si crystal) was used to correct the shifts in the pattern due to scanning of the beam across the map area. Results were filtered to exclude pixels with bad quality of cross-correlation indicated by normalized peak heights in the cross-correlation function <0.3 and pixels for which the displacement-gradient tensor was a poor fit to the measured shifts with mean angular error >0.004 radians (*52*). The mean angular error, to assess the quality of the analysis, and the HR-KAM, useful to evaluate the contribution of noise in the analysis, are reported in fig. S8.

Residual stresses were calculated from the measured elastic strains using Hooke’s law and considering elastic constants of garnet (*53*). Values of residual stress are relative to the stress state at the reference point used for the cross-correlation. As most of the selected reference patterns come from areas affected by some residual elastic strains, the components of the residual stress tensor were normalized to their mean values for every grain to reduce the effect of the choice of reference pattern on the measurements (*54*). Only the in-plane components of the stress tensor are presented because, given the assumption of plane-strain conditions required for the calculation of the strain matrix, the third dimension is not explorable with HR-EBSD. The direction perpendicular to the sample surface is also almost completely relaxed during sample preparation.

Probability plots are presented to shows characteristics of the stress distributions in the maps following an approach similar to that of Wallis *et al.* (*43*). Probability distributions are plotted considering a binwidth of 20 MPa. The distribution of normalized stresses is characterized by normal probability plots and the shape of the tails are studied with plots of the restricted second moments of the distributions (*35*, *42*). Normal probability plots are plotted with the cumulative-probability axis scaled such that a normal distribution falls along a straight line. Departures from a straight line, indicating a deviation from the same normal distribution, are typically associated with high stress magnitudes due to presence of dislocations. The restricted second moment (υ_2_) of the probability (*P*) distribution is calculated as the integral over restricted ranges of stress as υ_2_(σ) = $\int_{-\sigma }^{+\sigma }$*P*(σ)σ^2^*d*σ. A plot of υ_2_ versus ln(σ) is a straight line at high stress when the probability distribution has the form *P*(σ) ∝ ∣σ∣^−^^3^ as expected for the stress field generated by the presence of dislocations, and its gradient is proportional to the total dislocation density (*35*, *42*).

### Uncertainties in the calculation of *W*_CP_ from HR-EBSD data

As discussed above, HR-EBSD cannot measure rotations and strains in the third dimension (i.e., the dimension normal to the thin-section surface). For this reason, total GND densities and residual elastic strains are underestimated, affecting the calculation of *W*_CP_. Another potential cause of uncertainty in the calculation of *W*_CP_ is due to the fact that in large portions of the maps, GND densities are below the noise level (*27*). However, because *E*_disl_ is on average two orders of magnitude smaller than *E*_st_, the effect of local overestimates of GND density may be neglected. Statistically stored dislocations (SSDs) are also not measurable, but the measured residual elastic strains, being determined both by the presence of GNDs and SSDs, compensate the lack of a direct measure of SSDs. Another potential cause of uncertainty in the calculation is due to potential slight underestimation of σ_11_ or overestimation of σ_22_ that is inferable from the general dominance of σ_22_ over σ_11_. This trend is present in other HR-EBSD datasets and it is nontrivial to correct. However, for the order-of-magnitude calculation of *W*_CP_ that we performed here such uncertainty can be considered not relevant.

### Clast-size distributions from EBSD and high-resolution imaging

Four EBSD maps were acquired for grain-size analysis and were processed using MTEX. A threshold angle of 2° was used for the reconstruction to account also for small rotations between neighbor grains due to local shattering and the absence of shearing of the pulverized aggregates. Given the absence of subgrains in the garnets, the choice of a small misorientation angle is appropriate and does not affect the total count of grains. Grains with fewer than 7 pixels were discarded, limiting the measurement to a minimum equivalent radius of 0.22 μm.

Not indexed portions of the EBSD maps where investigated in three sites by high-resolution BSE imaging. Three images (3072 × 3072 pixels, field of view 25 μm, pixel size ~8.1 nm) where acquired in selected domains. Because of varying gray shades in the smaller grains (effect of nano-topographic imperfection and boundary effects), clasts were manually drawn on each image. The drawn clasts where then measured using the image analysis software ImageJ.

Log-log plots of clast size classes versus the cumulative number of particles per class were plotted for the four EBSD maps together and separately for the three images. The distributions were fit in log-log space with different segments with slope *D_i_*. A cumulative graph to compare the distributions was plotted by normalizing every class of clast size by the covered area. A value of *D* = 2.1 was estimated to approximate the entire clast-size distribution by averaging the *D* values of the segments covering the largest portions of the distributions (i.e., over the range 0.3 to 10 μm). The entire distribution was considered as fractal, considering that, because of limitations in the analysis method, the largest and smallest fragments may be underestimated (*15*).

### Calculation of fracture energy

Fracture energy was calculated from the clast-size distribution following the approach proposed by Pittarello *et al.* (*15*). Fracture energy was calculated as *W*_SF_ = *A*_SZ_γ, where *A*_SZ_ is the new surface area of clasts per unit area of fault produced in the slipping zone and γ is the specific surface energy, γ = 3.551 J/m^2^ (*55*).

The measured two-dimensional clast-size distribution was extrapolated to the corresponding three-dimensional distribution, transforming the calculated fractal coefficient *D* into D* (the three-dimensional fractal distribution coefficient) following the equation D* = *D* + *D*′, with *D*′ in the range 0 to 1 (*56*).

*A*_SZ_ was calculated following the approach proposed by Pittarello *et al.* (*15*). The total surface area in the investigated domain, *A*_SZ*i*_, was calculated from the clast-size distribution by

$$A_{\text{SZ}i}=\int_{r_{\text{min}}}^{r_{\text{max}}}4\pi r^{2}\mathrm{dN}\approx \frac{4\pi CD^{*}}{2-D^{*}}\left(r_{\text{max}}^{2-D^{*}}-r_{\text{min}}^{2-D^{*}}\right).$$ (5)

*A*_SZ*i*_ was then extrapolated to the volume of pseudotachylyte corresponding to a square meter of fault (*V*_SZ_ = *w* * 1m^2^ with *w* the thickness of the pseudotachylyte fault vein) by multiplying it by the ratio *V*_SZ_/*V*_SZ*i*_. *V*_SZ*i*_ is the volume of the fragments, approximated as spheres, of the investigated domain and was calculated from the clast-size distribution by

$$V_{\text{SZ}i}=\int_{r_{\text{min}}}^{r_{\text{max}}}\frac{4}{3}\pi r^{3}\mathrm{dN}\approx \frac{4\pi CD^{*}}{3\left(3-D^{*}\right)}\left(r_{\text{max}}^{3-D^{*}}-r_{\text{min}}^{3-D^{*}}\right)$$ (6)

### Heat calculation

The heat due to frictional heating during seismic slip per unit fault area can be calculated as *W*_FH_ = [*H*(1 − ϕ) + *c*_p_(*T*_m_ − *T*_hr_)]ρ*w*, with *H* the latent heat of fusion, *c*_p_ the specific heat at constant pressure, *T*_m_ the maximum temperature of the melt, *T*_hr_ the temperature of the host rock, ρ the rock density and ϕ the fraction of clasts in the pseudotachylyte vein (*25*). ϕ was obtained by measuring the area of the clasts in the pseudotachylyte that where identifiable in the thin-section scan. Smaller clasts scattered in the pseudotachylyte matrix and visible only with the scanning electron microscope were neglected. The garnet fraction was extracted from a BSE transect across the entire vein, manually excluding large garnet clasts. Plagioclase and biotite fractions were measured from high-resolution BSE images acquired in homogeneous portions of the pseudotachylyte matrix, excluding micrometric clasts (fig. S1). *T*_m_, the temperature of the melt, was assumed to be 1450°C in accordance with the temperatures proposed for superheated frictional melts in an upper crustal pseudotachylyte (*8*, *15*).

*c_p_* and *H* are calculated from values for pure minerals (*c*_p*i*_, *H_i_*) considering their relative abundance in the pseudotachylyte. Values of *c*_p*i*_ (1248, 1298, and 1347 JK^−1^ kg^−1^ for plagioclase, biotite and garnet respectively) were calculated for the given temperature conditions (maximum temperature of the melt, 1450°C) from the database of Holland and Powell (*57*), considering pure albite for plagioclase, phlogopite for biotite, and pyrope for garnet. Values of *H_i_* for silicates are commonly on the order of 500 kJ/kg (*58*). We used value of 302 kJ/kg for plagioclase and 453 kJ/kg for biotite from Di Toro and Pennacchioni (*8*) along with a value of 500 kJ/kg for garnet.

### Stress during dynamic fracture propagation

The dynamic stress field associated with the earthquake rupture propagation was responsible for the strong elastic deformation and development of GNDs in the lattice of host-rock minerals, in addition to cataclasis. However, it is worth remarking that the stresses measured by HR-EBSD do not correspond to the causative dynamic stresses. The calculated dynamic stress field is reported here to support the origin of the residual elastic strains/stresses measured by HR-EBSD during the stage of fracture propagation because the spatial gradients of the dynamic stresses and of the residual elastic stresses normal to the pseudotachylyte fault vein show consistent patterns. We use the asymptotic solution for an in-plane propagating fracture by Freund (*41*) to calculate the stress field surrounding the tip of a mode II fracture. In the two-dimensional reference cartesian coordinate system, the fracture is parallel to the *x* axis and propagates toward right.

Following Reches and Dewers (*7*), two different reference frames are considered to calculate the stress: (i) one centered at the fracture moving tip, where the stress is calculated at every point *P*(*r*,θ) with *r* the distance from the tip of the fracture and θ the angle; and (ii) the other fixed in space where the stress is calculated at a fixed point *P*(*x*,*y*) away from the passing fracture as a function of time *t* = *x*/*v*, with *v* the velocity of the fracture tip and *x* its position along the *x* axis.

The stress field is expressed as

$$\sigma_{\text{ij}}=\frac{K_{\text{II}}}{\sqrt{2\pi r}}\sum_{\mathrm{ij}}^{\mathrm{II}}\left(\theta ,v\right)$$ (7)

with $\sum_{\mathrm{ij}}^{\text{II}}\left(\theta ,v\right)$ representing the angular variations of the stress components for any values of the instantaneous crack tip-speed

$$\sum_{11}^{\text{II}}=-\frac{2\alpha_{s}}{D}\left[\left(1+2\alpha_{d}^{2}-\alpha_{s}^{2}\right)\frac{\text{sin}0.5\theta_{d}}{\sqrt{\gamma_{d}}}-\left(1+\alpha_{s}^{2}\right)\frac{\text{sin}0.5\theta_{s}}{\sqrt{\gamma_{s}}}\right]$$ (8)

$$\sum_{12}^{\text{II}}=\frac{1}{D}\left[\left(4\alpha_{d}\alpha_{s}\right)\frac{\text{cos}0.5\theta_{d}}{\sqrt{\gamma_{d}}}-{\left(1+\alpha_{s}^{2}\right)}^{2}\frac{\text{cos}0.5\theta_{s}}{\sqrt{\gamma_{s}}}\right]$$ (9)

$$\sum_{22}^{\text{II}}=\frac{2\alpha_{s}\left(1+\alpha_{s}^{2}\right)}{D}\left[\frac{\text{sin}0.5\theta_{d}}{\sqrt{\gamma_{d}}}-\frac{\text{sin}0.5\theta_{s}}{\sqrt{\gamma_{s}}}\right]$$ (10)

The parameters in the equations are

$$\alpha_{d}=\sqrt{1-v^{2}/C_{d}^{2}}$$ (11)

$$\alpha_{s}=\sqrt{1-v^{2}/C_{s}^{2}}$$ (12)

$$D=4\alpha_{d}\alpha_{s}-{\left(1+\alpha_{s}^{2}\right)}^{2}$$ (13)

$$\gamma_{d}=\sqrt{1-{\left(v\text{sin}\theta /C_{d}\right)}^{2}}$$ (14)

$$\gamma_{s}=\sqrt{1-{\left(v\text{sin}\theta /C_{s}\right)}^{2}}$$ (15)

$$\text{tan}\theta_{d}=\alpha_{d}\text{tan}\theta$$ (16)

and

$$\text{tan}\theta_{s}=\alpha_{s}\text{tan}\theta$$ (17)

where *C*_d_ and *C*_s_ are the velocity of compressional and shear waves. The solution is valid for fracture velocities below the Rayleigh-wave velocity, *C*_r_ = 0.92 *C*_s_.

*K*_II_ is the stress-intensity factor and determines the magnitude of the crack-tip stress field. For a fracture propagating at equilibrium conditions, *K*_II_ is related to the energy-release rate, *G*, and the Young modulus, *E*, by *G* = *K*_II_^2^/*E*. For a dynamic fracture, the relation depends upon the propagation velocity, and can be written as (*41*)

$$G=\frac{1-\upsilon }{2\mu }A_{\text{II}}K_{\text{II}}^{2}$$ (18)

where

$$A_{\text{II}}=\frac{v^{2}\alpha_{s}}{\left(1-\upsilon \right)C_{s}^{2}D}$$ (19)

We considered the following material properties: density ρ = 2800 kg/m^3^, shear modulus μ = 30 GPa and Poisson’s ratio υ = 0.22. The energy-release rate of a propagating fracture can be considered equal to the fracture energy *G*_c_ of the host material. We considered a value of *G*_c_ of 10^6^ J/m^2^ compatible with values typical of earthquake ruptures (10^5^ to 10^7^ J/m^2^) (*12*, *16*). The velocity of propagation of the fracture has a first-order effect on the magnitude of the tip stress field. For our simulations, we considered a velocity of 0.9*C_s_*, close to the Rayleigh velocity.
