# Supplementary material for: On-fault earthquake energy density partitioning from shocked garnet in an exhumed seismic midcrustal fault
Source: Sci Adv. 2024 Mar 1;10(9):eadi8533. doi: 10.1126/sciadv.adi8533 (PMC10906929; doi:10.1126/sciadv.adi8533)
Supplement: Supplementary file 1 — Figs. S1 to S8 [file sciadv.adi8533_sm.pdf]

Science Advances
AAAS

Supplementary Materials for

On-fault earthquake energy density partitioning from shocked garnet in an exhumed seismic midcrustal fault

Giovanni Toffol *et al.*

Corresponding author: Giovanni Toffol, giovanni.toffol@unipd.it

Sci. Adv. **10**, eadi8533 (2024)
DOI: 10.1126/sciadv.adi8533

This PDF file includes:

Figs. S1 to S8

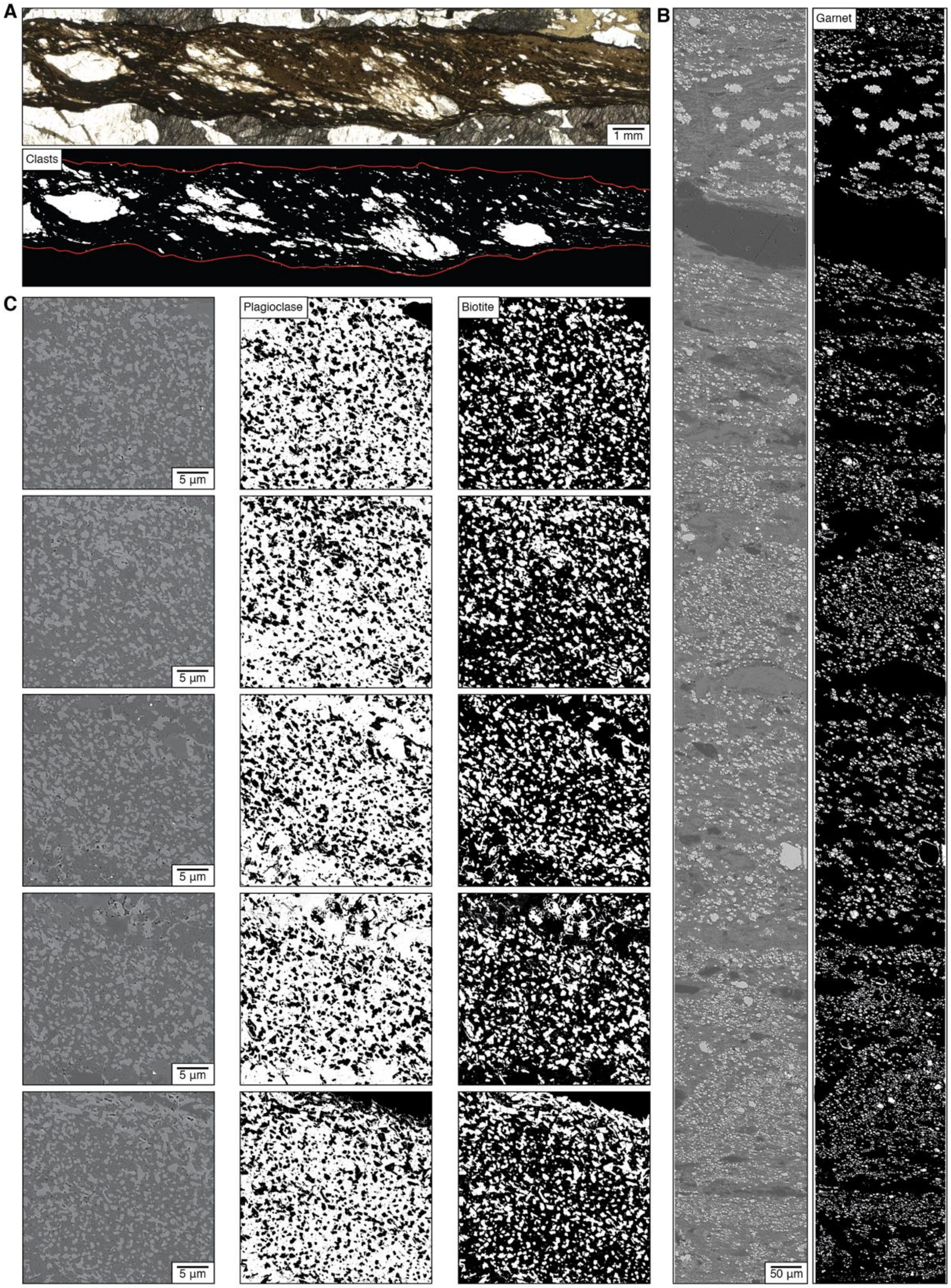

Fig. S1. Pseudotachylyte composition. (**A**), Pseudotachylyte fault vein and segmented clasts. (**B**), Backscattered electrons (BSE) transect across the pseudotachylyte and segmented garnet microlites. (**C**), High-resolution BSE images of the pseudotachylyte matrix and segmented plagioclase and biotite.

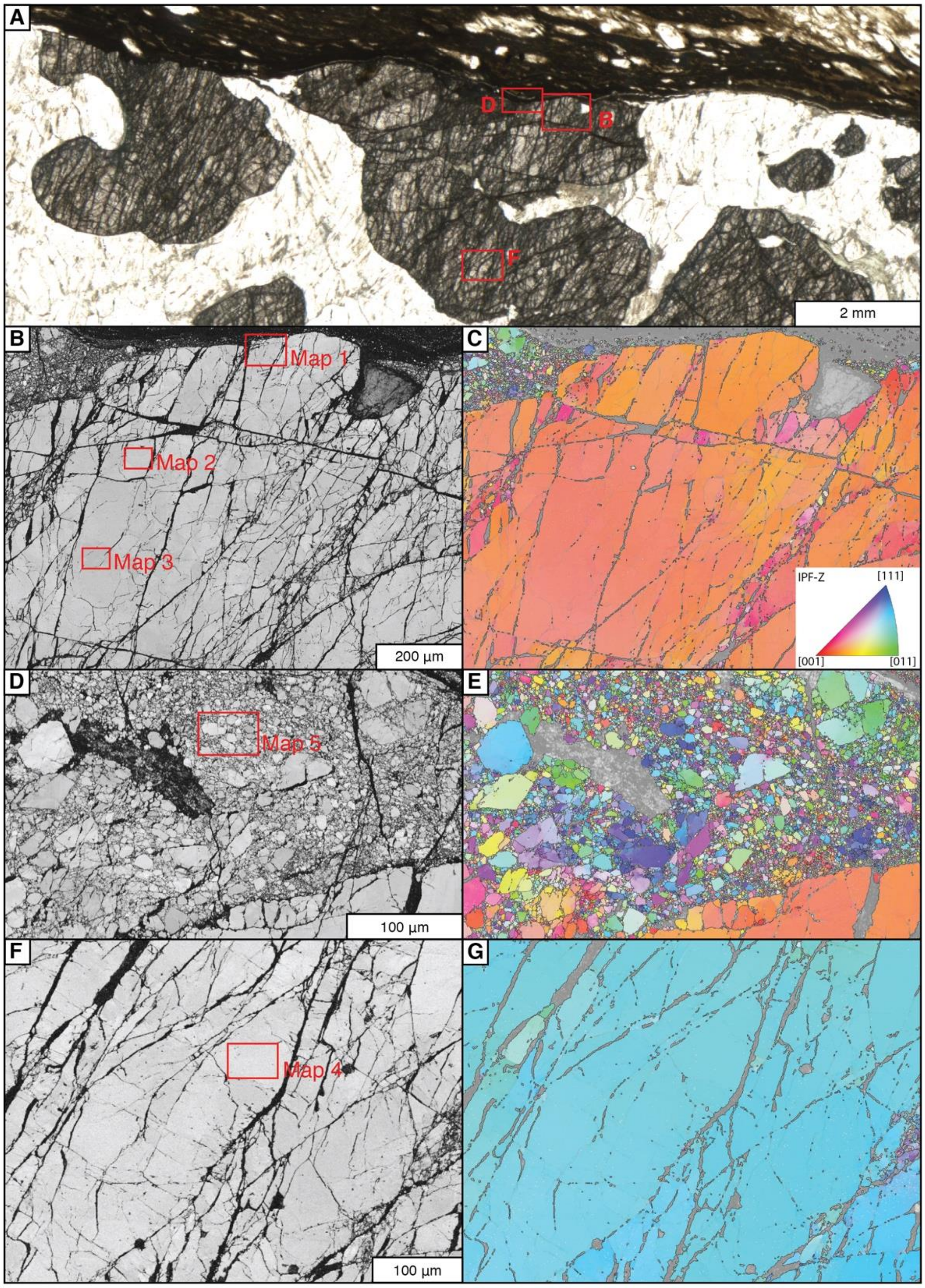

Fig. S2. High-angular resolution electron backscattered (HR-EBSD) maps locations. (**A**), Visible light image of the investigated garnets. Red squares mark the locations of the EBSD maps. (**B**, **D**, **F**), Band-contrast EBSD maps. Red squares mark the locations of the five HR-EBSD maps. (**C**, **E**, **G**), EBSD maps color-coded by crystal orientation according to the inverse pole figure inset in the bottom-right corner of (**C**) with respect to the Z direction

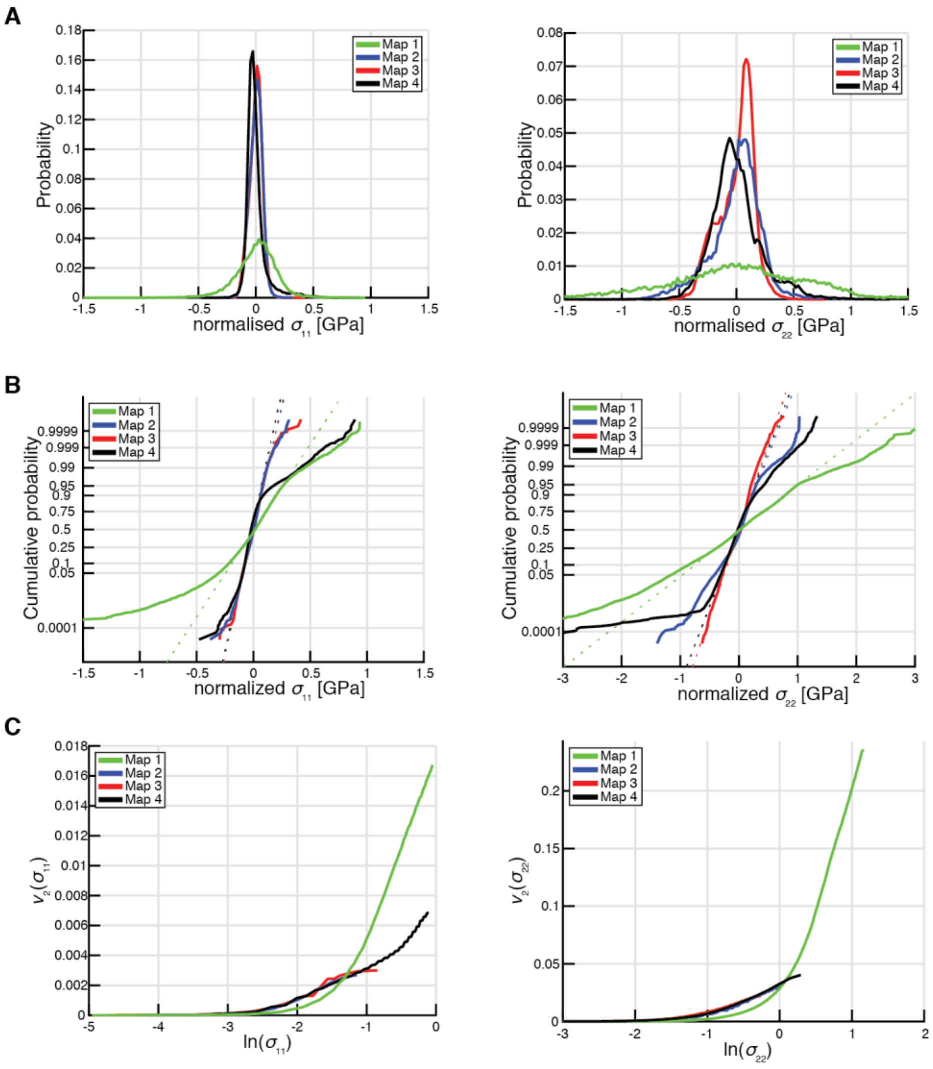

Fig. S3. Probability distribution and distribution form analysis of the stress heterogeneities for $\boldsymbol{\sigma_{11}}$ and $\boldsymbol{\sigma_{22}}$. (**A**), probability distribution. (**B**), normalized probability distribution. (**C**), restricted second moment of the probability distribution

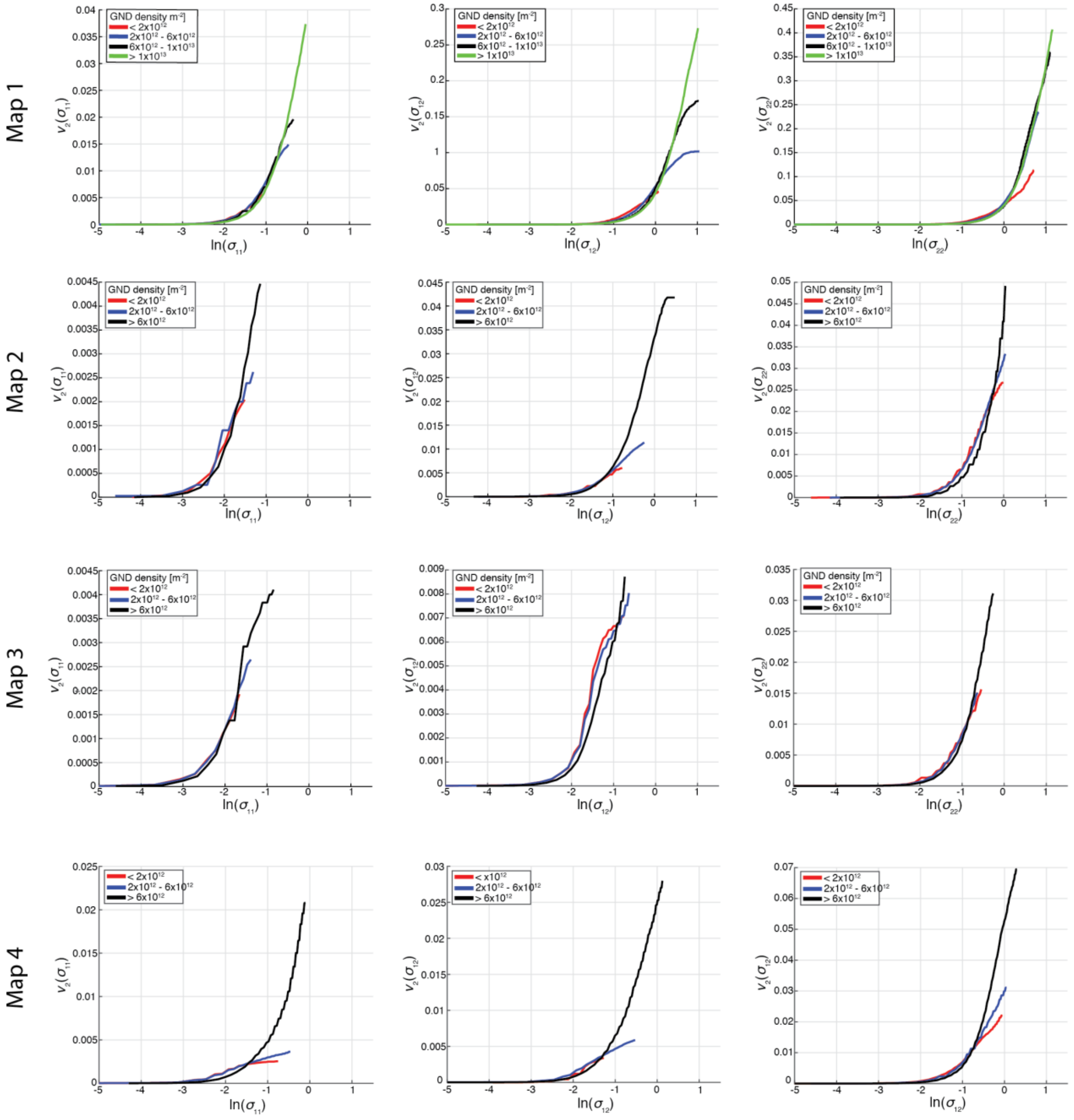

Fig. S4. Restricted second moments of the stress distributions for the three in-plane components of the residual stress. Different subsets are plotted for every map based on intervals of increasing geometrically necessary dislocation (GND) densities.

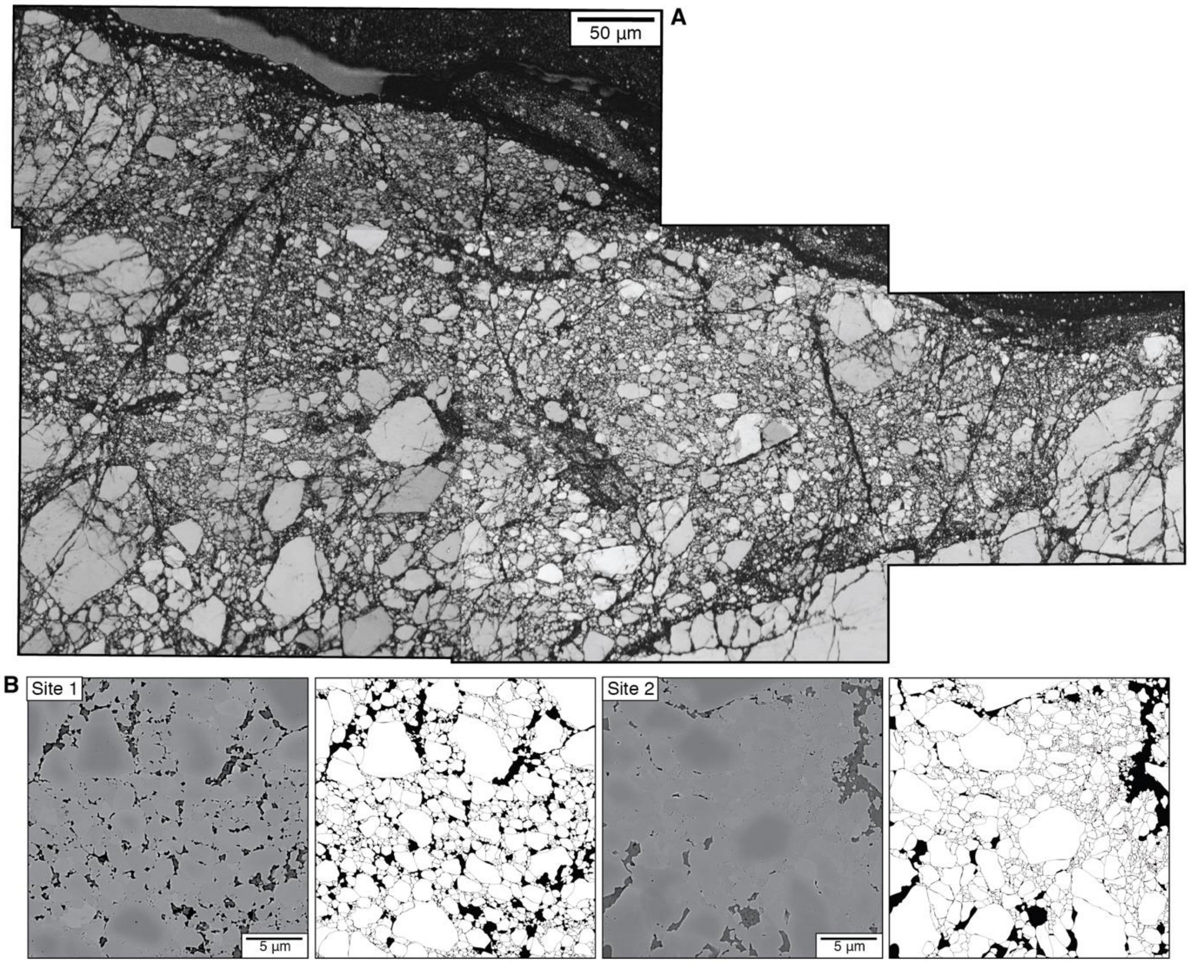

Fig. S5. Clast-size analysis. (**A**), electron backscattered diffraction (EBSD) band-contrast maps of the cataclastic domain considered for clast-size analysis and presented in Fig. 6. (**B**), High-resolution BSE images and segmented garnet grains for the Sites 1 and 2.

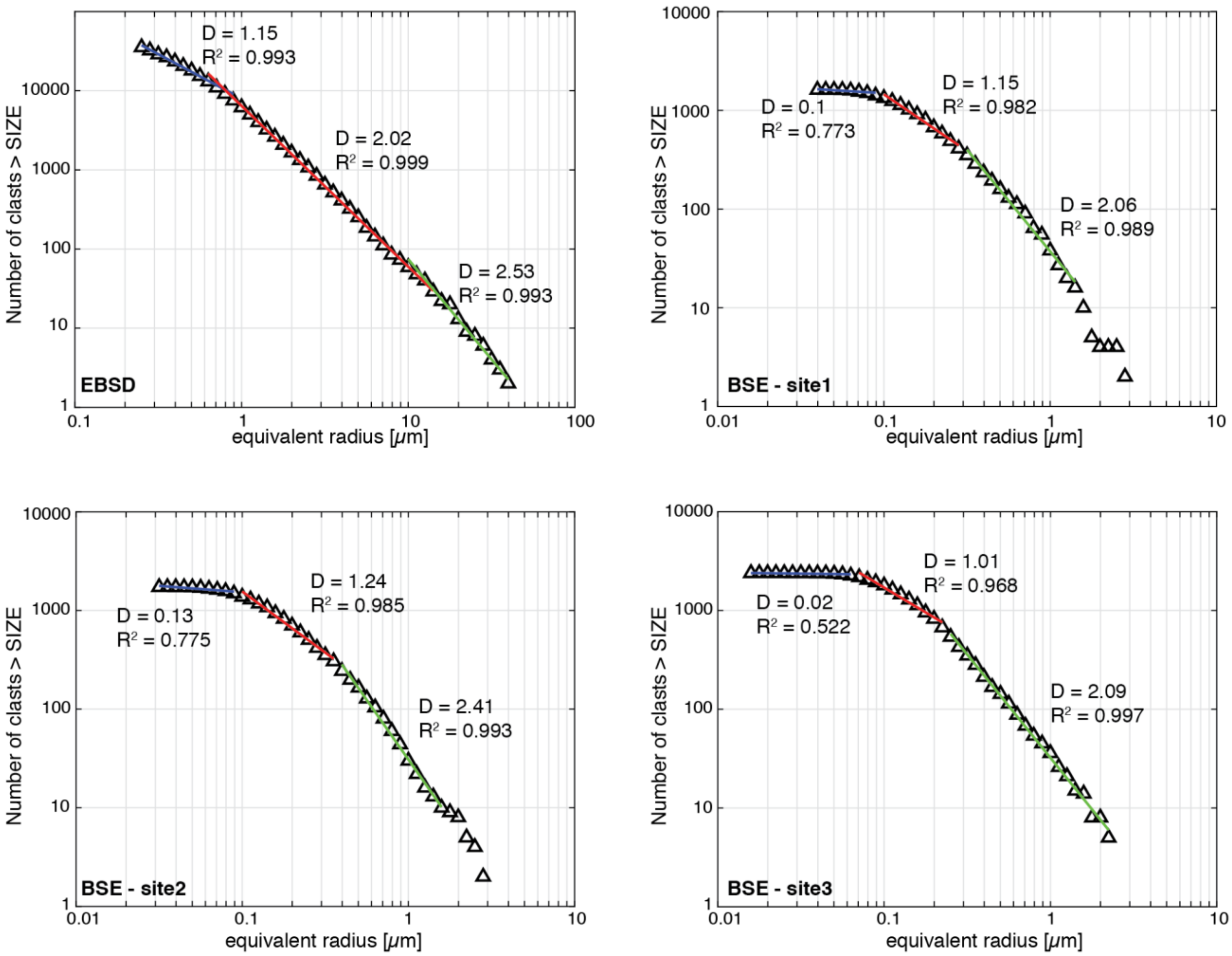

Fig. S6. Clast-size distribution graphs. Log-log graphs with equivalent radius in x-axis and cumulative number of clasts per class on the y-axis. Interpolating segments are represented with the respective D-values and R-squared fitting parameters.

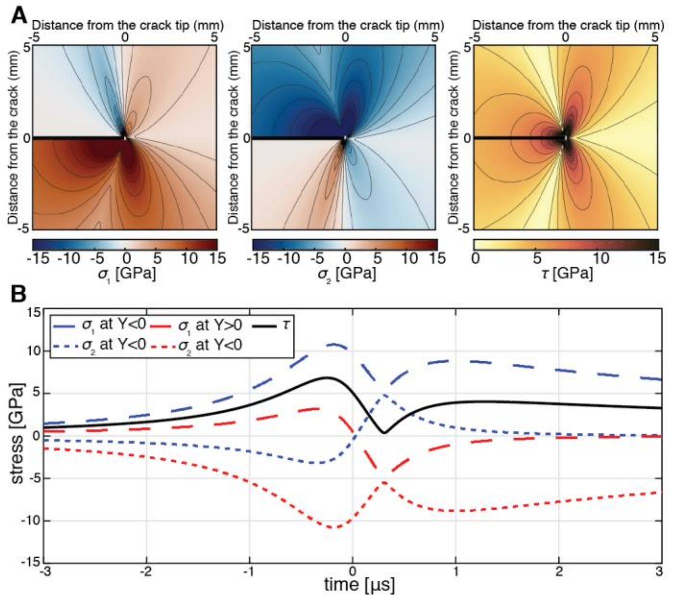

Fig. S7. Stress field surrounding the tip of a mode II propagating fracture. Stress components calculated for a fracture propagating at a velocity corresponding to 0.9 of the shear wave velocity, close to Rayleigh velocity (0.92 of the shear wave velocity) in a material with elastic properties typical of middle-crustal felsic rocks (see Methods). Calculations are performed in a reference frame moving with the tip of the fracture and in a fixed frame. (**A**), maximum principal stress, minimum principal stress and shear stress around the tip of a propagating fracture. The interval of stress contours is 1.5 GPa. (**B**), Stress plotted as function of time at a fixed point located at a distance of 3 mm (above, Y>0, and below, Y<0) from the rupture surface during fracture propagation. At t = 0 s, the fracture tip is at the shortest distance from the fixed point; the fracture tip is approaching the point and proceeding away from it at negative and positive values, respectively.

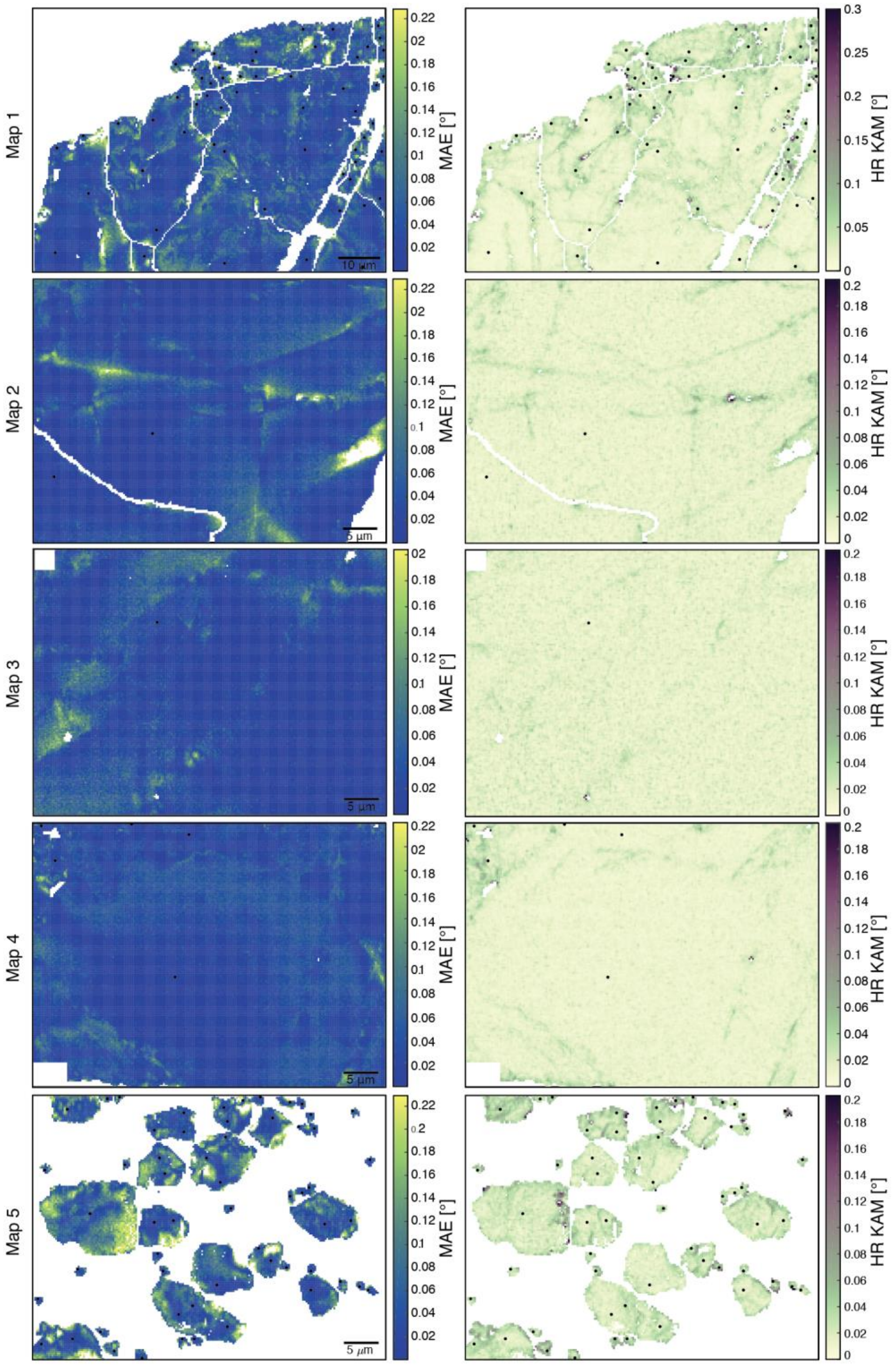

Fig. S8. Mean angular error (MAE) and high-resolution kernel average misorientation (HR-KAM). MAE is a parameter useful to evaluate the quality of the cross correlation. Only points with MAE below 0.2292° are considered reliable for the analysis. HR-KAM, calculated in every pixel as the average misorientation with respect to the surrounding pixels, is useful to visualize the noise contribution in the analysis.
